# Altered serum levels of IL-33 in patients with advanced systolic chronic heart failure: correlation with oxidative stress

**DOI:** 10.1186/1479-5876-10-120

**Published:** 2012-06-08

**Authors:** Hai-Feng Zhang, Shuang-Lun Xie, Yang-Xin Chen, Jing-Ting Mai, Jing-Feng Wang, Wa-Li Zhu, Li-Guang Zhu

**Affiliations:** 1 Department of Cardiology, Sun Yat-sen Memory hospital, Sun Yat-sen University, Guangzhou, China; 2 Department of Cardiology, Institute of Cardiovascular Diseases, First Affiliated Hospital, Guangxi Medical University, Nanning, China

**Keywords:** Chronic heart failure, Interleukin-33, Soluble ST2, Oxidative stress

## Abstract

**Background:**

Interleukin-33 (IL-33) has been linked to chronic heart failure (CHF) in animal studies, but data on serum IL-33 levels in human CHF are not available. We analyzed levels of IL-33 in serum, and investigated the possible role of IL-33 in oxidative stress.

**Methods:**

A total of 191 subjects with advanced systolic CHF (CHF group), 175 patients with pre-existing cardiac diseases but no CHF (non-CHF group), and 177 healthy controls (HC group) were enrolled. Serum levels of IL-33, soluble ST2 (sST2) and N-terminal-pro-brain natriuretic peptide (NT-proBNP), malondialdehyde (MDA) content, erythrocyte superoxide dismutase (eSOD) activity, as well as left ventricular ejection fraction (LVEF), were determined. The exact form of IL-33 in serum was identified. Effects of IL-33 and sST2 on MDA content and SOD activity in angiotensin (Ang II)-stimulated AC16 cells were assessed.

**Results:**

Serum levels of IL-33 and sST2 were elevated in CHF patients, whereas IL-33/sST2 ratios were decreased. In CHF patients, pre-existing cardiac diseases and medications used upon hospital admission did not affect IL-33 concentrations or the IL-33/sST2 ratio. Full-length IL-33, which could not be detected in serum from HC and barely detected in non-CHF patients, was significantly up-regulated in CHF patients. IL-33 levels were positively correlated with markers of CHF severity. IL-33/sST2 ratios were slightly and negatively related to MDA concentrations. IL-33 directly reduced MDA and enhanced SOD activity in Ang II-stimulated AC16 cells, which were greatly attenuated by sST2.

**Conclusions:**

Serum levels of IL-33, especially the full-length form, were elevated in CHF patients whereas IL-33 bioactivity was reduced. In advanced CHF, IL-33 may exert anti-oxidation effects, which may be overwhelmed by concurrently elevated levels of sST2.

## Background

Despite advances in treatment, chronic heart failure (CHF) remains one of the most important life-threatening cardiovascular conditions. It is associated with a high incidence in many western countries, poor outcome, and considerable healthcare costs [1,2]. CHF is a state of chronic deterioration in health with enhanced oxidative stress [3]. Positive correlations between increased levels of oxidative stress and CHF severity have been reported [3]. Moreover, therapy involving inhibitors of oxidation benefits CHF patients [4].

Interleukin (IL)-33 is an IL-1 superfamily cytokine [5,6]. It is the unique endogenous ligand for ST2 with soluble (sST2) and transmembrane (ST2L) isoforms, which are encoded by the same gene but regulated by different promoters, and which produce different messenger ribonucleic acid molecules (mRNAs) [7]. ST2L mediates the effects of IL-33 whereas sST2 limits the activity of IL-33 [8]. Alterations in the serum levels of sST2 in CHF have been examined in a wide range of clinical studies. The results from these large clinical trials have consistently shown that sST2 levels are elevated in CHF patients. Moreover, sST2 levels correlated with plasma concentrations of brain natriuretic peptide (BNP) and predicted adverse outcomes [9,10]. In contrast to many IL-1 superfamily cytokines (e.g., IL-1β and IL-18) which can have negative roles in cardiac diseases and ventricular remodeling [11], IL-33 has been demonstrated to have a potent protective role in various experimental CHF studies through inhibition of the nuclear factor kappa B (NF-κB) cascade and reduction of oxidative stress [8,12].

IL-33 expression is significantly up-regulated by mechanical strain in cardiomyocytes and cardiac fibroblasts [8]. Ventricular wall stress and mechanical load on ventricular muscle fibers is substantially increased in CHF patients, especially in those with end-stage CHF. Hence, we hypothesized that IL-33 levels may be increased in patients with advanced CHF and may play a part in the regulation of oxidative response in these patients. Given the important role of sST2 in CHF and IL-33 bioactivity, we determined sST2 levels and explored their interactions with IL-33, and in relation to oxidative stress.

## Methods

The study protocol was approved by the Ethics Committee of Sun Yat-sen Memorial Hospital (affiliated to Sun Yat-sen University, Guangzhou, China). Written informed consent was obtained from each participant or his/her relative/carer.

### Study population

One hundred and ninety-one consecutive patients with advanced systolic CHF (CHF group) admitted to Sun Yat-sen Memorial Hospital and the First Affiliated Hospital of Guangxi Medical University (Nanning, China) from 2009 to 2011 were included. CHF was diagnosed based on a history of cardiac diseases, symptoms and signs of cardiac diseases, echocardiographic results, and plasma levels of N-terminal-pro-brain natriuretic peptide (NT-proBNP) according to established criteria [13,14]. Major exclusion criteria were: (i) CHF with preserved left ventricular ejection fraction (LVEF ≥40%); (ii) acute heart failure, acute myocardial infarction and myocarditis; (ii) known current or past allergic diseases (e.g., asthma), autoimmune diseases (e.g., systemic lupus erythematous), inflammatory diseases (e.g., rheumatic arthritis and inflammatory bowel disease), or malignant diseases. One hundred and seventy-five age- and sex-matched patients with similar pre-existing cardiac diseases but without CHF (non-CHF group) were admitted in the same period and on the same hospital wards. In addition, age-, sex- and ethnicity matched healthy individuals were used as healthy controls (HC group). All participants were from the Chinese Han population. Strengthening the Reporting of Observational Studies in Epidemiology (STROBE) recommendations were used in the development of the population study [15].

### Culture and stimulation of cells

To evaluate the anti-oxidative effects of IL-33 in human cardiomyocytes, we assessed malondialdehyde (MDA) contents and superoxide dismutase (SOD) activity in angiotensin II (Ang II)-stimulated AC16 cells (ATCC, Bethesda, MD, USA), a cell line derived from the primary cultures of adult ventricular cardiomyocytes [16]. Cells were maintained and grown as described previously [16]. Briefly, cells were plated in a six-well plate at 0.3 × 10^6^/ml and incubated in Dulbecco’s modified Eagle’s medium nutrient mixture F-12 (DMEM-F12; Invitrogen, Carlsbad, CA, USA) with 1% penicillin (Invitrogen), 1% streptomycin (Invitrogen), and 12.5% FBS (Invitrogen) at 37°C and an atmosphere of 5% CO_2_ for 12 h. AC16 cells were stimulated with Ang II (10 nmol/L, Sigma-Aldrich, St. Louis, MO, USA) for 24 h in the presence or absence of recombinant human IL-33 (1 ng/ml, 5 ng/ml, 10 ng/ml, and 100 ng/ml; Sino Biological, Beijing, China) and sST2 (10 μg/ml; Sino Biological, Beijing, China).

### Biochemical measurements

Blood samples were obtained from all subjects by venipuncture upon hospital admission and collected in sterile non-treated tubes. Serum was obtained by centrifugation (2000 rpm for 5 min), immediately frozen, and stored at −80°C until subsequent batched analyses. Concentrations of IL-33 and sST2 in serum were analyzed using commercially available enzyme-linked immunoassay (ELISA) kits (IL-33: Enzo, Farmingdale, New York, USA; sST2: Medical & Biological Laboratories, Naka-Ku, Nagoya, Japan). Given the possible role of sST2 in limiting IL-33 activity, we calculated the ratio of IL-33 to sST2 to approximately evaluate the possible bioactivity of circulating IL-33 [17]. MDA contents were determined using an established and commercially available method (Beyotime, Nantong, China) [18,19]. For assessment of the level of erythrocyte superoxide dismutase (eSOD), erythrocytes were lysed and eSOD activities were determined using commercial kits (Beyotime). Blood samples for the assessment of NT-proBNP levels were kept in ethylenediamine tetra-acetic acid (EDTA) tubes at room temperature and measured by electrochemiluminescence immunoassay using a Roche Elecsys® system (Roche, Basel, Switzerland).

It is well known that IL-33 can circulate in two forms: full length and cleaved. IL-33 concentrations in serum were too low to be detected directly by western blotting, so we undertook immunoprecipitation analyses followed by western blotting as described previously to identify the exact form of IL-33 [20,21]. Briefly, serum were immunoprecipitated with mIL-33 antibody (1:100 dilution; Enzo). Protein A agarose beads were added to collect the IL-33-mIL-33 antibody complex. For western blotting, samples were analyzed by sodium dodecyl sulfate-polyacrylamide gel electrophoresis (SDS-PAGE) and blotted onto polyvinylidene fluoride (PVDF) membranes (Millipore, Bedford, MA, USA). Membranes were blocked and incubated with primary antibody (mIL-33 antibody at 1:1000 dilution; Enzo) and then secondary antibody (horseradish peroxidase (HRP)-conjugated anti-mouse IgG at 1:10,000 dilution; Jackson Laboratory, Bar Harbor, ME, USA). Visualization of immunoreactive human IL-33 was undertaken using enhanced electrochemiluminescence (ECL) reagents (Thermo Fisher Scientific, Rockford, IL, USA).

For determinations of MDA levels and SOD activity in AC16 cells, cells were lysed and supernatants collected. MDA contents and SOD activity were measured using the commercial kits described above.

### Statistical analyses

Normally distributed continuous data are expressed as mean ± SD or mean ± SEM (for MDA content and SOD activity in AC16 cells), otherwise as quartiles (first, median, and third). Categorical variables are presented as absolute and relative frequencies. Statistical comparisons across groups were made using Pearson’s χ^2^ test for categorical variables and the *t*-test (or one-way ANOVA followed by the Student-Newman-Keuls test for multiple *post-hoc* comparisons if three or more groups were compared) was used for normally distributed data. For non-normally distributed data, the Mann–Whitney test was used for two groups and the Kruskal-Wallis with Dunn’s *post-hoc* test was adopted for more groups. To test whether the statistical difference of IL-33 between the CHF group and non-CHF group was significant with the potential confounding factors under consideration, we used logistic regression to calculate the adjusted the *P* values for IL-33 and the IL-33/sST2 ratio between the two groups (responsive variables: CHF group or non-CHF group; explanatory variables: IL-33 or IL-33/sST2 ratio, age, heart rate, blood pressure, pre-existing cardiac diseases, medications, and serum creatinine levels). Spearman’s correlation coefficients were used to assess the correlations between IL-33 (or IL-33/sST2 ratios) and other continuous variables. All analyses were done using SigmaPlot ver11.0 (SPSS, Chicago, IL, USA) and statistical charts were made using OriginPro ver8.0 (OriginLab, Boston, MA, USA).

## Results

### Clinical characteristics

Table 1 lists the baseline characteristics of the subjects. Age and sex profiles were not significantly different among the three groups. Patients were in New York Heart Association (NYHA) class III (78 cases, 40.84%) or class IV (113 cases, 59.16%). As expected, echocardiographic abnormalities (reduced LVEF and increased size of the left ventricle) were found in all CHF patients, whereas these parameters were essentially normal in non-CHF patients and controls. Levels of NT-proBNP, creatinine and markers of oxidative stress were significantly higher in CHF patients. The prevalence of various pre-existing cardiac diseases was similar in non-CHF and CHF patients; the predominant disease was coronary heart disease, followed by hypertension, a concurrence of the two diseases, and cardiomyopathy.

**Table 1:** Characteristics of all participants

	**HC**	**non-CHF**	**CHF**	P^*^
	**n = 177**	**n = 175**	**n = 191**	
Age (years)	66.72 ± 5.73	66.98 ± 6.32	66.35 ± 5.91	0.60
Male (%)	102 (57.62%)	101 (57.71%)	109 (57.07%)	0.99
HR (beat/min)	78.46 ± 11.22	81.30 ± 14.58	72.17 ± 16.94	<0.01
SBP (mmHg)	115.10 ± 14.97	136.98 ± 29.04	127.74 ± 23.46	<0.01
DBP (mmHg)	71.97 ± 9.48	76.34 ± 11.96	71.01 ± 15.90	<0.01
Echocardiographic parameters				
LVEF (%)	NR	68.12 ± 7.14	27. 90 ± 4.09	<0.01
LVEDD (mm)	NR	47.60 ± 3.44	59.07 ± 5.82	<0.01
LVESD (mm)	NR	29.04 ± 3.16	37.88 ± 4.14	<0.01
Primary cardiac diseases				
CHD (%)	NR	103 (58.86)	103 (53.93)	<0.01
HBP (%)	NR	41 (23.43)	39 (20.42)	
CHD+HBP (%)	NR	31 (17.71)	35 (18.32)	
DCM (%)	NR	0 (0)	14 (7.33)	
Chemical markers				
serum creatinine (μmol/)	NR	73.48 ± 5.04	130.58 ± 84.50	<0.01
NT-proBNP (ρg/ml)	NR	88.89 (7.14-311.89)	5492.06 (187.21-18199.75)	<0.01
eSOD (U/mg Hb)	NR	1.41 ± 0.77	2.27 ± 1.11	<0.01
MDA (nmol/l)	NR	4.11 ± 1.25	4.69 ± 1.97	<0.01

Continuous data are the mean ± SD or median and range (for NT-proBNP). Categorical variables are presented as absolute and relative frequencies.

*, For categorical data, *P* values were derived by Pearson’s χ^2^ test; for continuous data, *P* values were calculated using the one-way ANOVA except in relation to NT-proBNP, for which the Kruskal-Wallis test was used.

NR, not relevant; HC, healthy controls; CHF, chronic heart failure; CHD, coronary heart disease; HBP, hypertension; DCM, dilated cardiomyopathy; HR, heart rate; SBP, systolic blood pressure; DBP, diastolic blood pressure; LVEF, left ventricular ejection fraction; LVEDD, left ventricular end-diastolic dimension; LVESD, left ventricular end-systolic dimension; NT-proBNP, N-terminal probrain natriuretic peptide; eSOD, erythrocyte superoxide dismutase; MDA, malondialdehyde.

### Serum levels of IL-33 and sST2, and the IL-33/sST2 ratio

**Figure 1:**
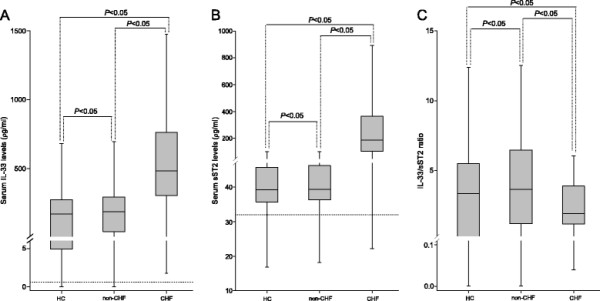
**Box chart for serum levels of IL-33 (A), sST2 (B), and IL-33/sST2 ratio (C).** Circulating IL-33 and sST2 were significantly increased in CHF patients whereas IL-33/sST2 ratios were decreased. Blood samples were obtained from healthy controls (HC, n = 177), cardiac-disease patients without CHF (non-CHF patients, n =175) and CHF patients (CHF patients, n = 191). Data are expressed as minima, quartiles, and maxima. Statistical analyses were done using the Kruskal-Wallis test followed by Dunn’s *post-hoc* test for multiple comparisons. The dotted line denotes the sensitivity of the ELISA kits. Abbreviations are the same as those shown in Table 1.

Serum levels of IL-33 and sST2 upon hospital admission were determined and compared among the three groups. Serum levels of IL-33 in the CHF group were significantly higher than those in non-CHF patients and healthy controls (Figure 1A). A similar trend was observed for the serum levels of sST2 (Figure 1B). Interestingly, despite elevated IL-33 concentrations, CHF patients showed significantly lower ratios of IL-33/sST2 compared with non-CHF patients and healthy controls (Figure 1C).

Some factors may affect IL-33 levels, so we used logistic regression analyses to calculate the *P* values of IL-33 and IL-33/sST2 ratio adjusted by age, heart rate, blood pressure, pre-existing cardiac diseases, medications, and serum creatinine levels. Serum IL-33 levels remained higher and IL-33/sST2 ratios remained lower after adjustments (for IL-33: *P* < 0.01; for IL-33/sST2 ratio: *P* < 0.01).

**Figure 2:**
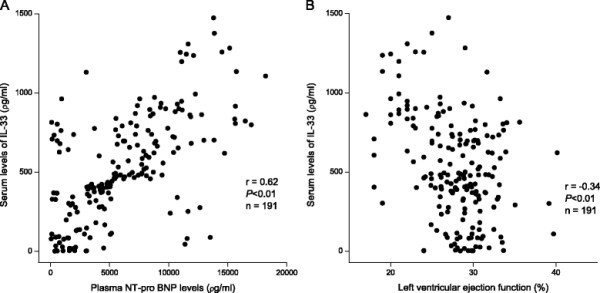
**Scatterplot between serum levels of IL-33 and NT-proBNP (A) and LVEF (B) in CHF patients.** Serum levels of IL-33 were significantly and positively correlated with those of NT-proBNP but negatively correlated with LVEF. Correlation coefficients were calculated by Spearman’s correlation analyses. Abbreviations are the same as those shown in Table 1.

To explore the potential correlations between IL-33 levels and CHF markers of severity, we performed Spearman’s correlation analyses to assess the relationships among IL-33 and NT-proBNP and LVEF. Results revealed a positive correlation between serum levels of IL-33 and NT-proBNP (Figure 2A) and a negative relationship between IL-33 concentrations and LVEF (Figure 2B).

### IL-33 forms

**Figure 3:**
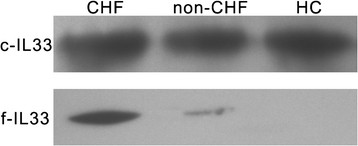
**Western blot analyses for serum in CHF patients, non-CHF patients, and HC.** IL-33 in serum was collected by immunoprecipitation, which was subsequently subjected to western blotting for analyzing the exact form of IL-33 in serum. Total IL-33 contents: ≈2.7 ng in CHF; ≈1.9 ng in non-CHF and ≈ 1.7 ng in HC. c-IL33, cleaved IL-33; f-IL33, full-length IL-33. Other abbreviations are the same as those shown in Table 1.

To identify the exact form of IL-33 in serum, we undertook immunoprecipitation and western blotting analyses. The full-length and cleaved form of IL-33 could be detected in CHF patients, whereas only the cleaved form was found in healthy controls. Full-length IL-33 was barely detected in non-CHF patients (Figure 3). We adopted all available methods to increase the sensitivity, but visual bands could be observed only in 15 CHF patients, 7 non-CHF patients, and 5 healthy controls. The limit of detection for immunoprecipitation and western blotting was ≈1.2-3 ng of total IL-33. These data suggested that full-length IL-33 was significantly increased in CHF patients.

### IL-33 levels and causes of CHF

**Figure 4:**
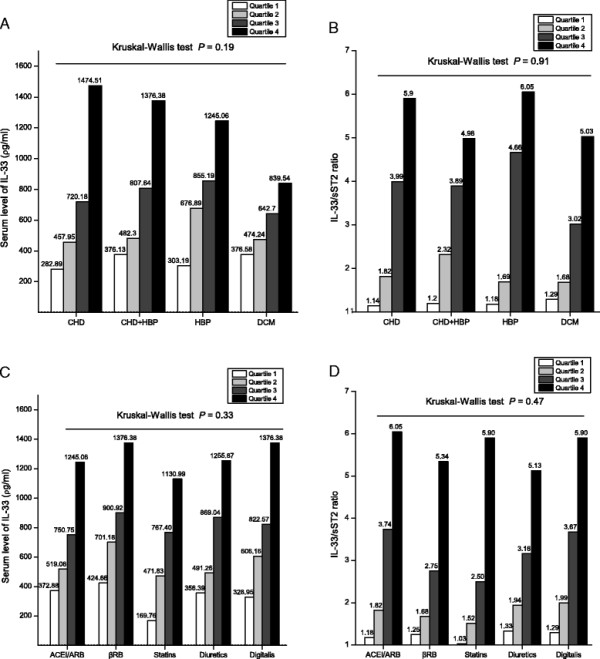
**Vertical bar chart for serum levels of IL-33 (A, C) and the IL-33/sST2 ratio (B, D) for various causes of CHF (A, B) and for different medications (C, D).** Serum samples were obtained from 103, 39, 35, and 14 patients with coronary heart disease, hypertension, coronary heart disease and hypertension, and dilated cardiomyopathy, respectively. Data are expressed as quartiles and maxima. Serum levels of IL-33 and IL-33/sST2 ratios were not significantly different among CHF patients with various causes (A, B). IL-33 levels and the IL-33/sST2 ratio in patients under different drug therapies was similar (C, D). Statistical analyses were done using the Kruskal-Wallis test. The dotted line denotes the sensitivity of the ELISA kits. Abbreviations are the same as those shown in Table 1.

To further explore whether different causes of CHF affected the parameters evaluated, we performed a subgroup analysis within the CHF group. Patients were divided into four groups according to the pre-existing cardiac disease causing CHF. The demographic data of patients in the four groups are shown in Table 2. Age, sex, echocardiographic data, and levels of NT-proBNP, creatinine and oxidation markers were not significantly different among the four groups. Results from the Kruskal-Wallis test did not show a significant difference in relation to serum levels of IL-33 or IL-33/sST2 ratios among groups (Figures 4A and B).

**Table 2:** Demographic data of patients with different causes of CHF

	**CHD**	**HBP**	**CHD+HBP**	**DCM**	P^*^
	**n = 103**	**n = 39**	**n = 35**	**n = 14**	
Age (years)	66.30 ± 5.99	64.77 ± 6.31	68.03 ± 5.51	66.93 ± 4.12	0.12
Male (%)	63 (61.17%)	20 (51.28%)	21 (60.00%)	5 (36%)	0.27
HR (beat/min)	70.78 ± 16.89	73.26 ± 15.89	72.97 ± 16.92	77.5 ± 20.19	0.51
SBP (mmHg)	117.40 ± 21.09	149.71 ± 10.67	144.94 ± 12.19	100.86 ± 3.10	<0.01
DBP (mmHg)	64.99 ± 14.36	84.69 ± 12.02	79.23 ± 12.80	58.14 ± 6.86	<0.01
NYHA III/IV	43/60	13/26	17/18	5/9	0.58
Echocardiographic data					
LVEF (%)	28.42 ± 4.04	27.48 ± 3.74	27.89 ± 4.11	28.47 ± 3.22	0.08
LVEDD (mm)	58.52 ± 6.40	59.53 ± 5.42	59.07 ± 5.82	61.32 ± 6.04	0.36
LVESD (mm)	37.53 ± 4.30	38.03 ± 3.94	37.88 ± 4.14	38.48 ± 4.49	0.60
Chemical markers					
serum creatinine (μmol/l)	126.70 ± 80.40	133.17 ± 93.37	137.06 ± 82.00	135.70 ± 101.50	0.92
NT-proBNP (ρg/ml)	5281.94 (239.83-19199.74)	5029.96 (187.21-15635.25)	6914.30 (687.84-15716.93)	5894.20 (1479.39-11550.13)	0.34
eSOD (U/mg Hb)	2.35 ± 1.10	2.22 ± 1.12	2.18 ± 1.24	2.04 ± 0.89	0.71
MDA (nmol/l)	4.90 ± 1.97	4.55 ± 1.93	4.15 ± 2.06	4.85 ± 1.79	0.25
Medications at arrival					
ACEIs/ARBs (%)	25 (24.27)	9 (23.08)	9 (25.71)	4 (28.57)	0.06
βRB (%)	10 (9.71)	8 (20.51)	9 (17.14)	1 (7.14)	0.08
Statins (%)	28 (27.18)	1 (2.56)	9 (25.71)	0 (0)	<0.01
Diuretics (%)	21 (20.29)	13 (33.33)	10 (28.57)	5 (35.71)	0.31
Digitalis (%)	25 (24.27)	12 (30.77)	10 (28.57)	2 (14.29)	0.61

Continuous data are the mean ± SD or median and range (for NT-proBNP). Categorical variables are presented as absolute and/or relative frequencies.

*For categorical data, *P* values were derived by Pearson’s χ^2^; for continuous data, *P* values were calculated using one-way ANOVA followed by the Student-Newman-Keuls test for multiple *post-hoc* comparisons except in relation to NT-proBNP, for which the Kruskal-Wallis test was used. NYHA, New York Heart Association; ACEI, angiotensin-converting enzyme inhibitor; ARB, angiotensin-II receptor blocker; β-RB, β-receptor blocker. Other abbreviations and acronyms are the same as shown in Table 1.

We also examined whether consumption of different medications upon hospital admission affected IL-33 levels. CHF patients were divided into five subgroups according to medications used upon arrival at hospital, and patients’ characteristics across different medication groups were similar (data not shown). Serum levels of IL-33 and IL-33/sST2 ratios were similar among different medical treatments upon hospital admission (Figures 4C and D).

### Levels of IL-33, sST2, the IL-33/sST2 ratio and markers of oxidation stress

**Figure 5:**
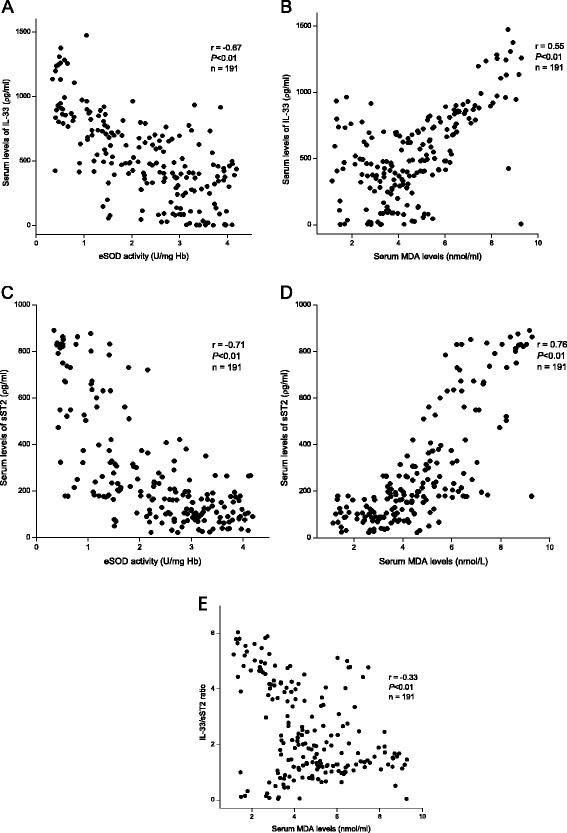
**Scatterplot between serum levels of IL-33 and eSOD activity (A) and MDA (B), serum levels of sST2 and eSOD activity (C) and MDA (D), and between IL-33/sST2 ratios and MDA levels (E) in CHF patients.** Serum levels of IL-33 were negatively correlated with eSOD activity and positively correlated with serum MDA contents (**A** and **B**). sST2 had a closer relationship with these oxidation markers (**C** and **D**). IL-33/sST2 ratios were slightly (but significantly) correlated with decreased MDA contents (**E**). Correlation coefficients were calculated by Spearman’s correlation analyses. Abbreviations are the same as those shown in Tables 1 and 2.

IL-33 has been reported to reduce ROS production [8]. We performed Spearman’s correlation analyses to determine whether serum levels of IL-33 correlated with eSOD activity and serum MDA content. Unexpectedly, IL-33 levels were negatively correlated with eSOD activity and positively related to serum MDA content (Figures 5A and B). sST2 had a closer relationship with these oxidation markers (Figures 5C and D). However, IL-33/sST2 ratios were slightly (but significantly) negatively correlated with serum MDA concentrations (Figure 5E). Previous experimental studies have shown that the anti-oxidation effects of IL-33 can be blocked by co-administration with sST2. Hence, these data may support the notion that the effects of IL-33 may be overwhelmed by concurrently elevated levels of sST2 in subjects with severe CHF.

### IL-33 directly attenuated oxidation stress *in vitro*

**Figure 6:**
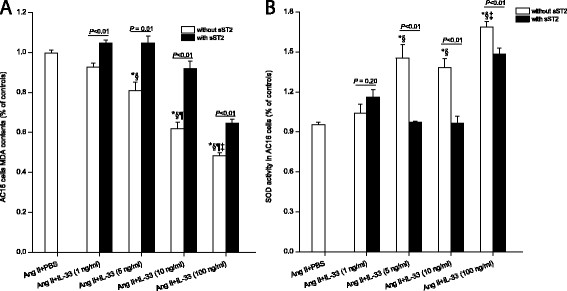
**Recombinant human IL-33 dose-dependently reduced MDA contents (A, n = 6) and increased SOD activity (B, n = 6) in Ang II-stimulated AC16 cells, which could be dramatically attenuated by sST2.** Data are the mean ± SEM. All results were from three independent sets. Statistical analyses were undertaken using one-way ANOVA with the Tukey *post-hoc* test for multiple comparisons. The paired *t*-test was used to compare values for samples with and without sST2 (10 μg/ml). *, *P* < 0.05 *versus* Ang II+PBS; §, *P* < 0.01 *versus* 1 ng/ml IL-33; ¶, *P* < 0.05 *versus* 5 ng/ml IL-33; ‡, *P* < 0.05 *versus* 10 ng/ml IL-33. Abbreviations are the same as those shown in Table 1.

To directly validate the antioxidative effects of increased levels of IL-33 in human cardiomyocytes, we assessed the effects of IL-33 in Ang II-stimulated AC16 cells and measured MDA content and SOD activity. IL-33 significantly increased SOD activity (Figure 6A) and reduced MDA levels (Figure 6B) in a dose-dependent manner. However, sST2 blocked these effects to a great extent (Figures 6A and B). These data suggested that sST2 could directly and considerably inhibit the antioxidative effects of IL-33 in human cardiomyocytes.

## Discussion

Since the discovery in 2005 that IL-33 as the natural ligand for ST2 [22], the IL-33/ST2 signalling pathway has been a focus of scientific interest. Some animal studies on this signalling pathway in CHF and cardiac fibrosis have been carried out [8,12] but data detailing IL-33 levels in human CHF have not been reported. The present study showed that serum levels of IL-33 were elevated in CHF patients whereas IL-33/sST2 ratios were significantly decreased, neither of which was associated with CHF aetiologies. Serum levels of IL-33 were positively correlated with levels of markers of oxidative stress, whereas the IL-33/sST2 ratio was associated with decreased MDA concentrations. We further confirmed that expression of full-length IL-33 was up-regulated in CHF patients. Moreover, we showed that IL-33 directly reduced MDA content and increased SOD activity in Ang II-stimulated AC16 cells, which however were overwhelmed by the presence of sST2.

IL-33 has been demonstrated to be protective in CHF, and the full-length form is thought to be more biologically active than the cleaved form [23]. Especially noteworthy was the suppression of NF-κB activity and the reduction of ROS by IL-33. Therefore, one may speculate that the significantly increased levels of the active form of IL-33 in CHF patients may have protective effects in the pathophysiological processes of CHF and may reduce oxidative stress. However, IL-33 levels were correlated with elevated levels of NT-proBNP, reduced LVEF, and markers of oxidative stress in these patients, which seemed to challenge the hypothesis of a protective role of IL-33 in CHF patients. One plausible explanation for these contradictory data may be that, as suggested in a previous study, the effects of IL-33 were blocked by concurrently elevated levels of sST2, which may serve as a decoy receptor and inhibit IL-33 bioactivity. Thus, the increased concentrations of IL-33 in CHF patients, despite being positively correlated with CHF severity and markers of oxidative stress, may have a compensatory role, which was limited due to the elevated levels of sST2. As a result, the elevation of IL-33 levels in CHF might only reflect disease severity and the extent of oxidative stress. The positive correlations between IL-33 levels and markers of oxidative stress may also be a result of inhibited bioactivity by sST2. This leads to an inability to decreased reduction of ROS [8] and subsequent oxidative stress. Notably, as suggested by other authors, the role of sST2 as a reservoir for IL-33 and increasing the circulation half-life of IL-33 (which has been found in the IL-6 receptor) cannot be ruled out [8,24].

In contrast to our findings, Dhillon and colleagues reported that IL-33 levels were not correlated with adverse outcomes in non-ST-elevation myocardial infarction (NSTEMI) patients [25]. Two phenomena may explain these conflicting findings. Firstly, the study by Dhillon *et al*. focused on the predicted value of IL-33 in NSTEMI patients, whereas the present study concentrated on the difference in IL-33 levels between CHF patients and non-CHF subjects. All the participants in the study by Dhillon *et al.* were NSTEMI patients, and comparisons between patients and healthy individuals were not made. Secondly, the studied population between the two studies was different. Few patients (13 of 577; 2.25%) suffered from pre-existing heart failure and only a few patients had heart failure (52 of 577; 9%) during the studying period in the study by Dhillon *et al*., whereas patients with acute myocardial infarction were excluded in our study. Therefore, the main diseases affecting serum levels of IL-33 in the previous study may have been myocardial infarction, whereas the main disease affecting serum IL-33 levels in the present study was severe systolic CHF. In addition, the mean value of LVEF in patients of the study by Dhillon *et al*. was much higher than that in the present study [25]. Therefore, the distinct characteristics of patients in the two studies may be an important reason for the different results. Interestingly, in the present study, IL-33 levels were positively correlated with NT-proBNP levels, a strong predictor for adverse outcome, which might indicate a role for IL-33 in predicting the adverse outcomes of CHF. IL-33 is not associated with poor outcome in NSTEMI patients [25], so the predictive value of IL-33 in CHF remains elusive.

The potential sources for the increased levels of IL-33 are unknown, and the release of full-length IL-33 is controversial [26,27]. Some researchers have reported elevated levels of IL-33 in serum and speculated that some cells (e.g., fibroblasts, endothelial cells, macrophages) may be the potential sources [28-30]. One study showed that IL-33 expression in cardiac fibroblasts was up-regulated upon mechanical strain [8]. Importantly, increased IL-33 contents were found in the supernatants of stimulated cardiac fibroblasts, which suggested that IL-33 could be secreted by cardiac fibroblasts [8]. Very recently, Lee and colleagues demonstrated that full-length IL-33 could be secreted directly by living fibroblasts upon biomechanical overload, which was noted in both mouse fibroblasts cell lines and human primary skin fibroblasts [31]. Therefore, one may speculate that mechanical stress in cardiac fibroblasts in CHF may be responsible (at least in part) for the elevated circulating levels of IL-33. In addition, endothelial cells have been shown to abundantly express IL-33 and increased endothelial damage has been found in CHF patients [32]. Thus, full-length IL-33 from the damaged endothelium may also contribute to the elevated serum levels of IL-33 observed in the present study. Even though studies have shown that cellular expression of IL-33 is increased upon stimulation [8], we found that IL-33 was scarcely detected in the supernatants of PMA-stimulated AC16 cells, whereas significantly increased levels of IL-33 could be found in cell lysates (data not shown). Thus, release of IL-33 from cardiomyocytes should be demonstrated in further studies, and more studies may be needed to explore the potential sources of IL-33 in CHF.

Some limitations of the current study should be noted. Firstly, all CHF patients recruited in the present study had advanced systolic CHF. Therefore, the importance of altered IL-33 levels in other CHF patients, especially in those with preserved ejection fraction, who have been demonstrated to have lower sST2 levels compared with those with systolic CHF [33], are not clear. Given the similar biomechanically induced secretion mechanisms shared by sST2 and IL-33 [8], one could hypothesize that IL-33 levels in CHF patients with preserved ejection fraction may be different. Secondly, all patients included in the present study were treated with anti-CHF therapies (e.g., β-receptor blockers, diuretics, angiotensin-converting enzyme inhibitors and angiotensin-II receptor blockers). Therefore, it may be difficult to compare our patients with other CHF patients who did not receive similar medications. Thirdly, as mentioned above, we did not explore the predictive value of IL-33 levels in CHF. Finally, even though some studies have used the IL-33/sST2 ratio to reflect IL-33 bioactivity, a change in the ratio might simply reflect differences in the mechanisms of induction of IL-33 and sST2, and may not necessarily be an indicator of IL-33 activity.

## Conclusions

The present study revealed significantly up-regulated circulating levels of IL-33 (especially the full-length form), but decreased IL-33/sST2 ratios in CHF patients. The IL-33/sST2 ratio was negatively correlated to oxidation in CHF patients. IL-33 directly attenuated oxidative stress, which was blocked by sST2. More studies are required to confirm these findings in CHF patients with preserved ejection fraction.

## Abbreviations

IL-33: Interleukin-33; CHF: Chronic heart failure; NT-pro BNP: NT-pro-brain natriuretic peptide; LVEF: Left ventricular ejection fraction; LVESD: Left ventricular end-systolic dimension; LVEDD: Left ventricular end-diastolic dimension; ROS: Reactive oxygen species; MDA: Malondialdehyde; eSOD: Erythrocyte superoxide dismutase; Ang II: Angiotensin II; CHD: Coronary heart disease; HBP: Hypertension; DCM: Dilated cardiomyopathy.

## Competing interests

The authors declare that no competing interests.

## Authors’ contributions

Jing-Feng Wang, Shuang-Lun Xie, Hai-Feng Zhang, and Li-Guang Zhu designed the study. Hai-Feng Zhang, Jing-Ting Mai performed the experiments. Hai-Feng Zhang and Yang-Xin Chen performed the statistical analyses. Hai-Feng Zhang, Shuang-Lun Xie, and Yang-Xin Chen drafted the manuscript and Jing-Feng Wang help to explain critical points in the manuscript. Wa-Li Zhu helped to collect samples and data. All authors read and approved the final manuscript.
